# JMJD3 upregulates ALOX5 to drive malignancy and concomitant ferroptosis sensitivity in gastric cancer

**DOI:** 10.1038/s41419-025-08020-1

**Published:** 2025-11-03

**Authors:** Gege Shu, Jiaoyang Yang, Huifang Hu, Anqi Dong, Tao Chen, Weikang Li, Xiaotong Sun, Peiyuan Li, Pengbo Wang, Changshun Shao, Jin Zhou

**Affiliations:** 1 Department of General Surgery, The First Affiliated Hospital of Soochow University, Suzhou, 215006 Jiangsu China; 2 Institute of Molecular Enzymology, School of Biology & Basic Medical Sciences, Suzhou Medical College of Soochow University, Suzhou, 215123 China; 3 Institutes for Translational Medicine, State Key Laboratory of Radiation Medicine and Protection, Suzhou Medical College of Soochow University, Suzhou, 215123 Jiangsu China

**Keywords:** Gastric cancer, Prognostic markers

## Abstract

Chemotherapy remains the cornerstone of gastric cancer (GC) treatment, with Oxaliplatin (OXA) being a critical first-line agent. However, chemotherapy resistance, compounded by increased stemness, poses a significant challenge in GC management. In this study, we demonstrate that JMJD3, encoded by *KDM6B* and catalyzing the demethylation of H3K27me3, is highly expressed in both GC tissues and patient-derived chemotherapy-resistant xenograft (PDX) models and contributes to increased malignancy and chemoresistance. Overexpression of JMJD3 enhanced stemness and chemoresistance in GC cells, while JMJD3 knockdown had opposite effects. Mechanistically, JMJD3 promotes GC cell stemness and chemoresistance by reducing H3K27me3 on the *ALOX5* promoter, a histone modification associated with ALOX5 transcriptional activation. Tumorigenesis induced by N-methyl-N-nitrosourea (MNU) was reduced in mice with gastric epithelial cell-specific deletion of *Kdm6b*. Importantly, ALOX5 upregulation due to the elevated JMJD3 function sensitized GC cells to ferroptosis inducers. These findings suggest that JMJD3 plays a pivotal role in GC chemoresistance by modulating both stemness and ferroptosis sensitivity. Targeting JMJD3 may provide a novel therapeutic strategy for overcoming chemotherapy resistance, with ferroptosis inducers potentially offering a promising adjunctive treatment in GC.

## Introduction

Gastric cancer is a prevalent malignancy within the digestive system. In 2020, it was estimated that there were approximately 1.08 million new cases of gastric cancer, constituting 5.6% of all malignant tumors, with an associated death toll of 769,000, accounting for 7.7% of deaths related to malignant tumors [1–3]. This disease exhibits high molecular and phenotypic heterogeneity [4, 5]. Due to its subtle and often undetectable symptoms, a significant proportion of gastric cancer patients are diagnosed at an advanced stage, missing the opportunity for surgical intervention. As a result, OXA-based chemotherapy remains the primary first-line option for the management of gastric cancer [6]. However, the long-term use of chemotherapy drugs often leads to chemoresistance, and the prognosis of patients will deteriorate dramatically after chemoresistance [7].

Despite the plethora of available chemotherapeutic regimens, their efficacy remains unsatisfactory, therefore, solving chemotherapy resistance in gastric cancer is urgently needed. The concept of cancer stem cells (CSCs) was introduced over 30 years ago [8]. When tumor cells acquire stem cell-like characteristics, their capacity for self-renewal and heterogeneity significantly increases, leading to enhanced resistance to chemotherapy and targeted therapies [9]. This, in turn, results in tumor recurrence and metastasis, significantly reducing the overall survival (OS) of patients. Gastric cancer stem cells (GCSCs) are capable of inhibiting apoptosis caused by chemotherapeutic drugs through the downregulation of pro-apoptotic proteins, such as Bax, Bak, and Puma, and the upregulation of anti-apoptotic proteins, such as Bcl-2, BCL-XL, and Mcl-1 [10]. GCSCs also possess strong DNA damage repair capabilities, facilitating the repair of chemotherapy-induced DNA damage [11]. During chemo- and radiotherapy, CSCs can evade cell death by entering a dormant state. Furthermore, studies have shown that the proportion of CSCs within tumors increases after multiple rounds of chemotherapy [12], further emphasizing the need to understand the mechanisms of chemoresistance from the perspective of stemness of gastric cancer.

Cancer cells resistant to conventional chemotherapy may become more susceptible to ferroptosis [13]. The combined use of ferroptosis inducers with conventional therapies, such as chemotherapy [14], targeted therapy [15], and radiotherapy [16], shows significant potential in overcoming chemotherapy resistance. Some studies have implicated ferroptosis, a form of regulated cell death triggered by iron-dependent lipid peroxidation, in the regulation of chemoresistance. Exosomal MTTP from adipocytes has been to inhibit ferroptosis and thus enhance chemoresistance in colorectal cancer [17]. However, the indications for the use of ferroptosis inducers and their role in overcoming chemotherapy resistance in gastric cancer have yet to be fully explored. Importantly, previous studies have identified ALOX5 as a key mediator of ferroptosis in various cancers, including melanoma and bladder cancer [18, 19], suggesting that ALOX5 expression may serve as an indicator of susceptibility to ferroptosis.

Histone lysine methylation emerges as a pivotal player in gene activation and suppression [20]. The trimethylation of lysine-27 on histone H3 (H3K27me3) stands out as a crucial epigenetic modification that is regulated by Jumonji domain containing protein 3 (JMJD3) and other enzymes [21]. JMJD3, functioning as a histone demethylase, is induced under inflammatory, viral, and carcinogenic stimuli, specifically removes methyl groups at H3K27 [22, 23]. Prior investigations have illuminated the significant role of JMJD3 in the initiation, development, and metastasis of diverse tumors [24]. High expression of JMJD3 is associated with poor prognosis and increased susceptibility in gastric cancer [25, 26]. However, the oncogenic mechanisms of JMJD3 in gastric cancer remains unexplored.

In this study, we report the function of JMJD3 in gastric cancer progression and chemotherapy resistance. JMJD3 drives the demethylation of the ALOX5 promoter, leading to its overexpression, which helps maintain gastric cancer stem cell properties and chemoresistance. However, the ALOX5 upregulation concomitantly renders gastric cancer sensitive to ferroptosis inducers. Our findings indicate that chemoresistant gastric cancer with ALOX5 overexpression may be targeted for induction of ferroptosis.

## Materials and methods

### Patient samples

A sum of 90 pairs of gastric cancer and adjacent non-cancerous tissues were collected between 2015 and 2017 in The First Affiliated Hospital of Soochow University. After the operation, some tissue samples were directly placed in liquid nitrogen and some tissue samples were fixed with paraformaldehyde and then were embedded by paraffin, all tissues were kept there for future use. The First Affiliated Hospital of Soochow University’s ethical committee examined and authorized this study, and all of the patients gave their informed permission in line with the Declaration of Helsinki and its subsequent version (approval number: #2022164).

### Cell lines and cell culture

Cell lines, including GES-1, AGS, BGC-823, HGC-27, MKN45, NCI-N87, SGC-7901 and SNU-1 were purchased from Procell Life Science & Technology Co., Ltd. (Wuhan, China) or Beyotime Biotechnology Company (Shanghai, China) in 2023 with STR certificate. The cells were all cultured in RPMI 1640 supplemented with 10% fetal bovine serum (Gibco, USA), 1% penicillin (Gibco, USA), and 1% streptomycin (Gibco, USA). The complete culture medium for these cells was maintained in a cell culture incubator at 37 °C with 5% CO_2_.

### Cell transfection

Plasmids containing the wild-type JMJD3 gene fragment, sh-JMJD3 sequence, wild-type ALOX5 gene fragment, sh-ALOX5 sequence and corresponding empty vectors (Shanghai Yanji Biomedical Technology Co., Ltd, China) were transfected into the respective cell lines as shown in the experimental results. All transfection experiments in this study were performed using Lipofectamine 3000 reagent (Invitrogen, USA), following the protocol provided (Carlsbad, https://tools.thermofisher.cn/content/sfs/manuals/lipofectamine3000_protocol.pdf). Puromycin was used to select cells transfected with the plasmid and to establish stable cell lines.

### Immunohistochemistry

A universal two-step detection kit (Mouse/Rabbit Polymer Detection System, ZSGB-BIO, PV-9000, China) was used for immunohistochemical experiments. The specific experimental procedures followed the protocol of the detection kit. The primary antibodies used were JMJD3 antibody (Abcam, ab38113, UK) at a concentration of 5 µg/ml and Ki-67 at a concentration of 2 µg/ml (Abcam, ab15580, UK).

### Quantitative reverse transcription polymerase chain reaction

The experimental method of qRT-PCR followed our previous research, and the mRNA primers used in this research were listed in Table S1.

### Western blot

The experimental method and procedure of WB were as previously reported [27], and the antibody dilution concentrations are listed in Table S2.

### Cell line-derived xenografts

Six-week-old male immunodeficient nude mice were selected as hosts for the nude mouse xenograft model. Cells (HGC-27-shCtrl, HGC-27-shJMJD3, AGS-shCtrl, AGS-sh-JMJD3, SNU-1-Vector, SNU-1-JMJD3, NCI-N87-Vector, NCI-N87-JMJD3) in exponential growth phase were suspended in PBS at a ratio of 100 μM PBS per 2 × 10^6^ cells and hypodermic injected into nude mice. After one week, OXA (OXA) were injected into peritoneum at a 5 mg/kg/d dose, GSK-J4 (MedChemExpress, HY-15648B, USA) were injected into peritoneum at a 1 mg/kg/d dose. Follow-up experimental treatment is shown in the experimental results.

### Patient-derived tumor xenograft model

Fresh gastric cancer tumor tissues were immediately placed in 4°C PBS, and within 2 h, they were implanted subcutaneously on the ventral side of M-NSG mice (Shanghai Model Organisms, China). The tumors were cut into 3 × 3 × 3 mm pieces, dipped evenly in a mixture of matrigel and culture medium, and transplanted. The growth of tumor xenografts was monitored daily, and when the tumors reached 5 mm × 5 mm, measurements and weights were recorded. The tumor tissue from the xenograft was termed P0 PDX. After reaching 800 ~ 1000 mm3, the mice were euthanized by cervical dislocation, and the tumor tissue was collected for subsequent passages (P2 and P3 PDX). Each generation of PDX could be fixed and frozen for experimental needs. Maximum dose of OXA was injected intraperitoneally with 2 mg/kg/d. All animals were kept in a specific pathogen-free environment. The experimental protocols for animal use were authorized by the Laboratory Animals Ethics Committee of Soochow University (Approval No.: SUDA20210916A05).

### Colony formation assay

Cells were seeded into 6-well plates at a density of 500–1000 cells per well and cultured under standard conditions for 10–14 days. Colonies were fixed with 4% paraformaldehyde for 15 min and stained with 0.1% crystal violet for 20 min. Visible colonies (≥50 cells) were imaged and counted using ImageJ software. Each group was assessed in triplicate.

### Sphere formation assay

Single-cell suspensions were plated in ultra-low attachment 96-well plates at a density of 500 cells per well in serum-free DMEM/F12 medium supplemented with B27, EGF (20 ng/mL), and bFGF (20 ng/mL). After 10–14 days of culture, primary spheres were collected using a 70 μm cell strainer, dissociated into single cells using trypsin, and reseeded under the same conditions for secondary sphere formation. Sphere formation efficiency (SFE) was calculated as the number of spheres >75 μm divided by the total number of seeded cells per well. Experiments were performed in triplicate.

### Organoid culture

After surgery, fresh gastric cancer tumor tissues were immediately placed in 4 °C BioGenous^TM^ primary tissue storage solution (K601005, USA) then sent to the laboratory. BioGenous^TM^ tumor tissue digestion solution (K601003, USA), organoid basal medium (B213152, USA), Matrigel (M315066, USA) and cancer organoid complete medium were used to establish organoid system (Please refer to https://www.biogenous.cn for detailed methods).

### CUT& TAG-seq and CUT& RUN

Tri-Methyl-Histone H3 (Lys27) (C36B11) Rabbit mAb #9733 (CST, USA) was used at a concentration of 1:50 for chromatin immunoprecipitation. DNA extraction kit (Hyperactive Universal CUT&Tag Assay Kit for Illumina Pro, TD904-01, Vazyme Biotech Co.,Ltd, China; Hyperactive pG-MNase CUT&RUN Assay Kit for PCR/qPCR, HD101-01, Vazyme Biotech Co.,Ltd, China) were used. The modified DNA is subsequently converted into a library and amplified through PCR. Next, the amplified DNA was detected by qPCR and was subjected to sequencing on a high-throughput platform. The sequencing data is then aligned to corresponding genomic locations using alignment tools, followed by the identification of DNA cleavage sites through Peak Calling tools. Finally, the experimental results are presented in a visually accessible format using visualization tools, facilitating an intuitive display of the data.

### Flow cytometry

1 × 10⁶ cells were harvested and resuspended in 100 μL of 1× PBS in a 15 mL centrifuge tube. A negative control was prepared using an identical cell suspension. In a separate tube, 2 mL of CD133 antibody (Abcam, 1:200, USA) was added to the cell suspension, followed by incubation in the dark at room temperature for 30 min. After incubation, the cells were washed with 1× PBS, centrifuged at 250×g for 5 min, and the supernatant was discarded. The cell pellet was resuspended in 400 μL of 1× PBS, filtered, and analyzed using a flow cytometer.

### JMJD3 cKO mice

*Tff1*-cre mice have been shown to specifically knock out genes in gastric epithelium using the Cre-LoxP system [28]. We purchased *Tff1*-cre mice from Shanghai Model Organisms (Shanghai, China). *KDM6B*^flox/flox^ mice were kindly provided by Dr. Xiaoren Zhang [29–31]. We crossed *KDM6B*^flox/flox^ to *Tff1*-cre mice to generate *KDM6B*^flox/flox^;*Tff1*-cre mice.

### Determination of ROS, malondialdehyde (MDA), and glutathione (GSH) within gastric cancer cells

Intracellular ROS levels were detected by confocal microscopy using the fluorescent probe DCFH-DA (Beyotime, S0033S, China). The Malondialdehyde (MDA) Assay Kit (Solarbio, BC0025, China) and the Reduced Glutathione (GSH) Assay Kit (Solarbio, BC1175, China) were used to analyze the production of MDA and GSH in GC cells.

### Statistical analysis

Data analysis was done using GraphPad Prism 6 (GraphPad software version 6.01). The clinicopathological characteristics uses the chi-square test. For each assay, the data were presented as the means ± standard deviation of at least three independent experiments. The Student’s *t*-test was used to examine the statistical significance. Kaplan-Meier method was used for analysis of overall survival (OS). *P* < 0.05 was regarded as significant.

## Results

### JMJD3 is upregulated in gastric cancer tissues, correlated with chemotherapy resistance and predicts poor prognosis

**Fig. 1:**
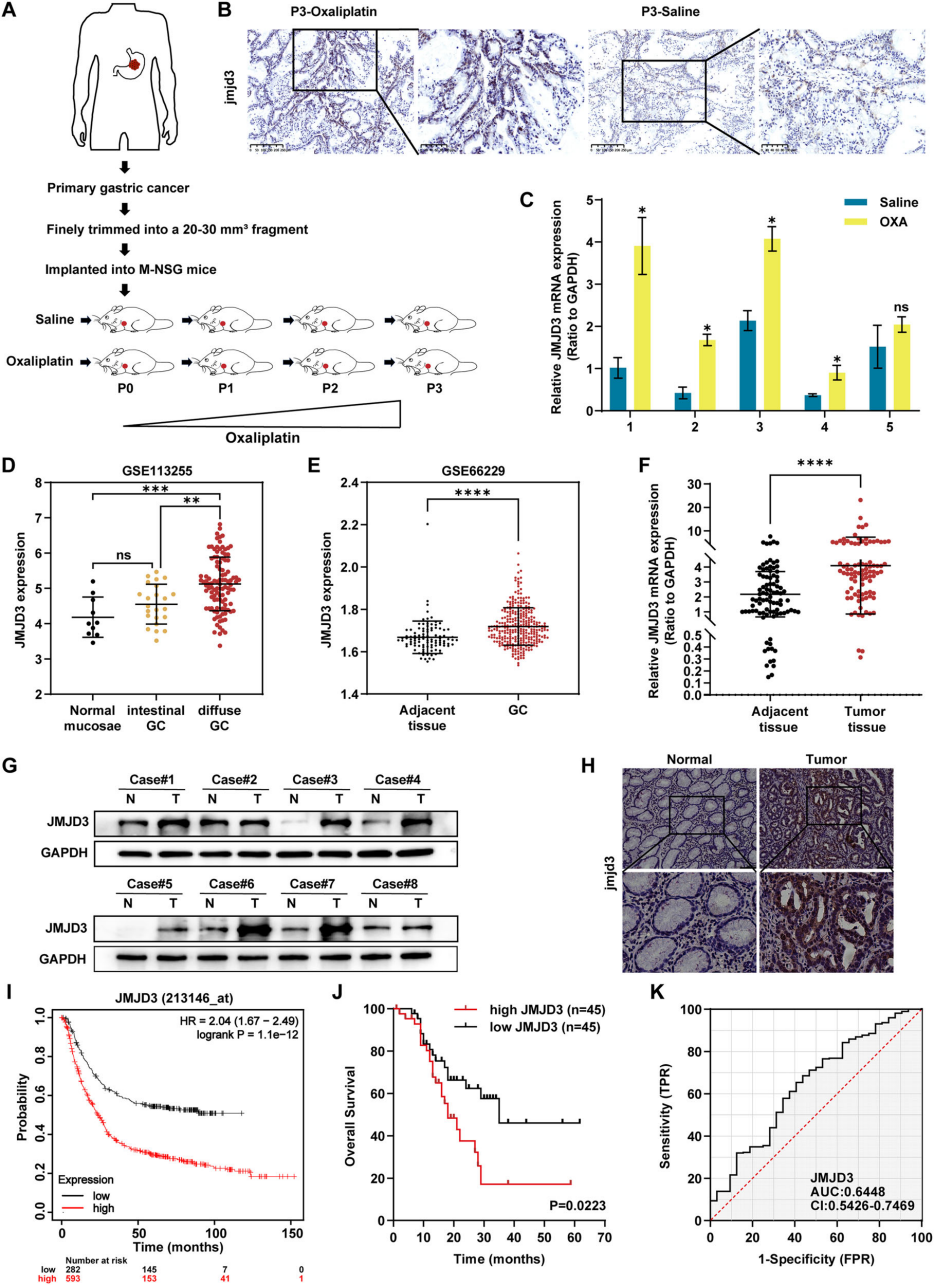
JMJD3 is upregulated in gastric cancer and predicts shorter survival. **A** Schematic representation of the OXA-treated-patient-derived xenograft (PDX) approach. **B** Representative images of immunohistochemistry staining for JMJD3 in tumor tissue from PDX treated with control or OXA. **C** mRNA expression of JMJD3 detected by qRT-PCR in tumor tissue from PDX treated with control or OXA. Multiple paired t tests were performed. **D** and **E** Gene expression of JMJD3 in human gastric cancer compared to normal tissues from 2 GEO data sets. **F** JMJD3 expression in gastric cancer tissues compared with paired adjacent tissues in 90 patients. WB (**G**) and Immunohistochemistry (**H**) revealed that JMJD3 was up-regulated in gastric cancer tissues compared to adjacent tissues. Scale bars = 75 µm. **I** Kaplan–Meier analysis of the OS rates in patients with gastric cancer presenting high or low expression of JMJD3 using data from TCGA. **J** Kaplan-Meier analysis indicating overall survival of gastric cancer patients with high (red) (*n* = 45) or low (black) (*n* = 45) JMJD3 expression. **K** ROC curve of JMJD3 expression in the diagnosis of gastric cancer. T tests were performed. The data are the means ± S.D. of three independent experiments. *p < 0.05; **p < 0.01; ***p < 0.001; **** *p* < 0.0001; ns no significance.

The development of resistance to OXA generally takes place within a few weeks following the beginning of treatment, thereby limiting its overall efficacy as a first-line chemotherapy for gastric cancer [32]. An oxaliplatin (OXA)-treated PDX model was established to identify the associated proteins (Fig. 1A). Surgically resected primary gastric cancer tissue was meticulously trimmed and directly implanted into M-NSG mice. The mice were randomly assigned to different groups and treated with either saline (control) or OXA in each generation. Previous studies have indicated that abnormal histone demethylation can occur during gastric cancer development [33]. To investigate the relationship between OXA resistance and histone demethylation, we examined the expression of several histone demethylases, including JMJD1A, JMJD2A, JMJD2B, JMJD2C, JMJD2D, JMJD3, and JMJD6, in patient-derived xenograft (PDX) models. Five independent PDX models (numbered 1–5, passage 3) were established from gastric cancer patients, and each model was divided into two treatment groups receiving either OXA or saline. As shown in Fig. 1C and S1A, JMJD3 expression was significantly elevated in OXA-treated groups compared with their corresponding saline-treated controls. mRNA expression data were collected from tumor tissues harvested from at least three mice per treatment group. These findings were further confirmed by immunohistochemical analysis (Fig. 1B). Additionally, high expression levels of *JMJD3* were observed in two Gene Expression Omnibus (GEO) datasets (GSE113255 and GSE66229) of gastric adenocarcinoma (STAD) compared to their corresponding normal tissues (NT) (Fig. 1D, E). This was corroborated by qRT-PCR, Western blotting (WB), and immunohistochemistry, which showed higher levels of JMJD3 in tumor tissues compared to adjacent normal tissues (Fig. 1F, G, H). Furthermore, elevated JMJD3 expression was associated with poor clinical outcomes in gastric cancer (Fig. 1I, J). The correlation between JMJD3 levels and clinicopathological characteristics, including tumor size, TNM staging, and Helicobacter pylori infection, was also observed (Supplementary Table S3). Figure 1K shows the ROC curve analysis, with an area under the curve (AUC) of 0.6448, indicating significant clinical value in identifying gastric cancer. Collectively, our findings from both PDX models and human datasets suggest that JMJD3 may function as an oncogene in gastric cancer and is associated with chemoresistance to OXA.

### JMJD3 inhibits the chemosensitivity of gastric cancer cells to OXA

**Fig. 2:**
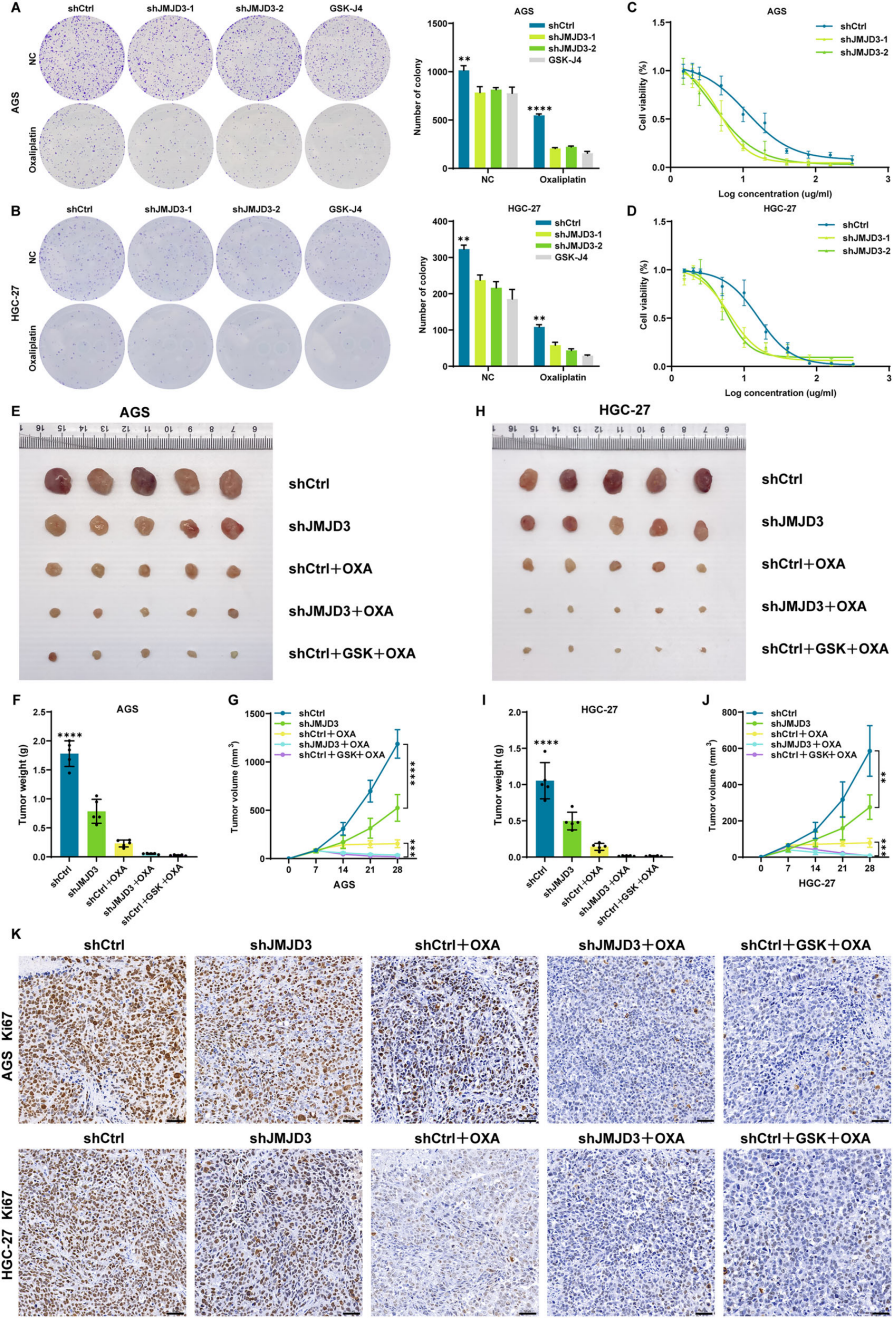
JMJD3 suppresses the chemosensitivity of gastric cancer cells to OXA. **A**, **B** Colony formation in shJMJD3 or shCtrl transfected gastric cancer cells then treated with OXA/GSK-J4. Multiple unpaired t tests were performed. **C**, **D** Dose response curves of OXA in AGS and HGC-27 cells expressing the indicated vectors. **E**, **H** Representative images of xenograft tumors in nude mice after different treatments. OXA were injected intraperitoneally daily for 3 weeks. The combination of shJMJD3 with OXA had stronger inhibitory effects on tumor growth. **F**, **I** The tumor weight of each group of mice are summarized. Ordinary one-way ANOVA were performed. **G**, **J** The tumor growth curves of each group of mice are summarized. Multiple unpaired t tests were performed. **K** IHC staining for Ki67 expression in different groups. Scale bars = 50 µm. The data are the means ± S.D. of three independent experiments. **p < 0.01; ***p < 0.001; ****p < 0.0001.

Due to the increase in JMJD3 expression in chemoresistant PDX models, we examined whether JMJD3 could affect the chemosensitivity of gastric cancer cells. JMJD3 expression was evaluated across various gastric cancer cell lines relative to the normal gastric epithelial cell line GES-1. JMJD3 was highly expressed in most gastric cancer cell lines compared with GES-1 (Supplementary Fig. S1B, C). Among them, AGS and HGC-27 cells exhibited markedly elevated JMJD3 expression, whereas SNU-1 and NCI-N87 cells showed relatively lower JMJD3 levels. Therefore, we selected these four cell lines to perform JMJD3 knockdown and overexpression experiments, with manipulation efficiency validated by Western blotting and qPCR (Supplementary Fig. S1D, E). Gastric cancer cells were sensitized to OXA through ectopic suppression of JMJD3, as evidenced by a decrease in colony formation ability and IC50 for OXA (Fig. 2A–D). However, when JMJD3 was overexpressed, the outcome was exact opposite (Supplementary Fig. S2A-D). The statistical analysis revealed that the decrease of JMJD3 exhibited a synergistic impact when combined with OXA. A xenograft tumor model was then developed by injecting nude mice with AGS and HGC-27 cells subcutaneously to examine the effects of JMJD3 knockdown on chemosensitivity. Seven days after cancer cell inoculation the tumor-bearing nude mice were split into 5 groups (n = 5 per group) and were intraperitoneally injected with GSK-J4, a dual inhibitor of H3K27me3/me2-demethylases JMJD3, or OXA. Silencing the expression of JMJD3 resulted in significant suppression of tumor growth in comparison to the Vec groups. The inhibitory effect of shJMJD3 in combination with OXA on tumor growth was more pronounced compared to that of any individual treatment (Fig. 2E–J). Additionally, weak staining for Ki67 was observed when JMJD3 knockdown was combined with OXA treatment (Fig. 2K). Collectively, these results showed that silencing JMJD3 enhanced the susceptibility of gastric cancer cells to OXA chemotherapy.

### JMJD3 confers gastric cancer cells stem cell-like characteristics

**Fig. 3:**
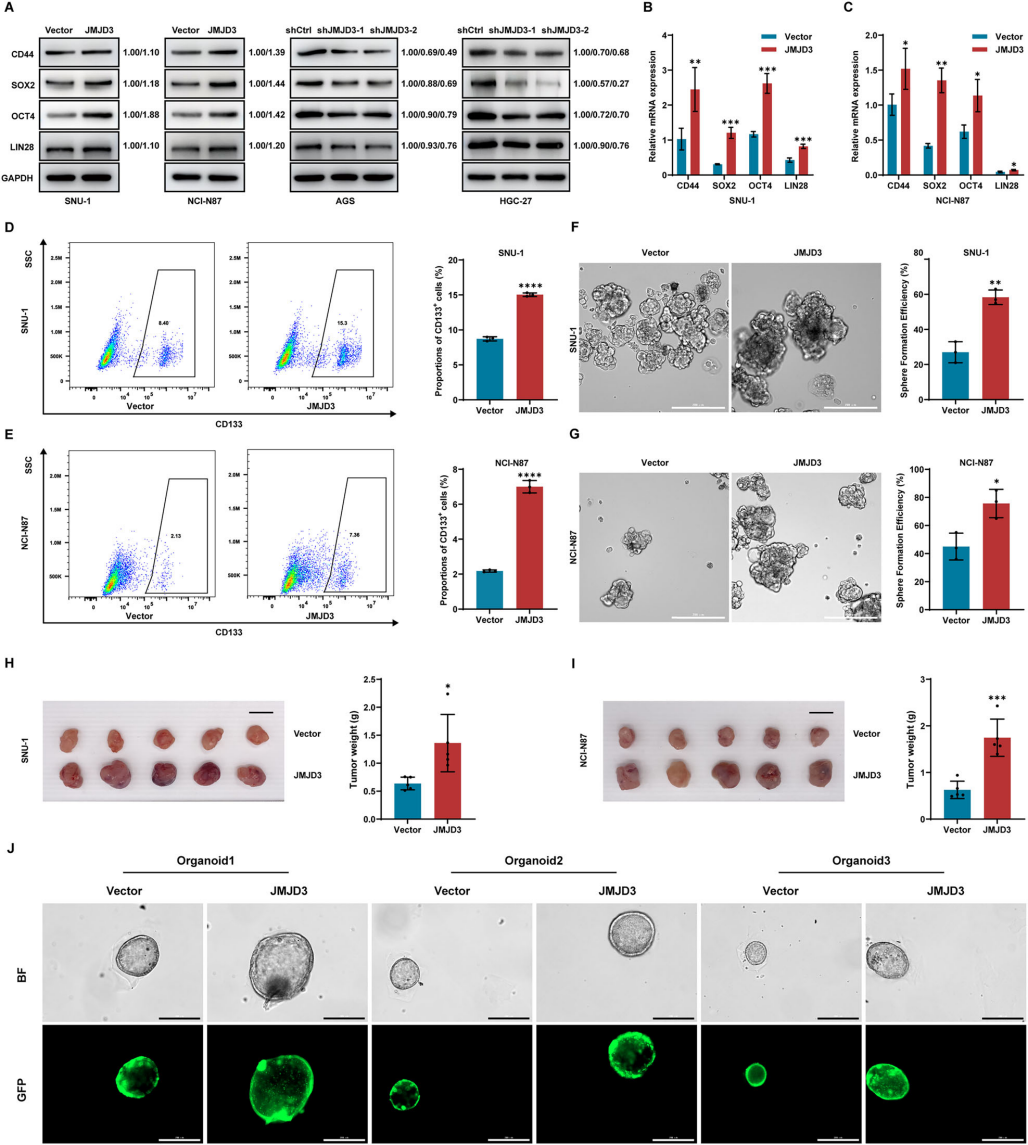
JMJD3 maintains the stemness of gastric cancer cells. **A** The protein expression levels of CSC markers, including CD44, SOX2, OCT4 and LIN28, were examined in shJMJD3-transfected gastric cancer cells and JMJD3 overexpression plasmid-transfected gastric cancer cells by western blot. **B**, **C** The mRNA expression levels of CSC markers. Multiple unpaired t tests were performed. **D**, **E** Flow cytometry was used to assess the percentage of CD133^high^ cells in gastric cancer cells with JMJD3 overexpression. Unpaired t tests were performed. **F**, **G** Tumor sphere formation assay were used to detect the proliferation rate of SNU-1 and NCI-N87 cells after transfected with indicated plasmid. Scale bars = 200 µm. Unpaired t tests were performed. **H**, **I** Nude mice were injected subcutaneously with cells and tumor formation monitored over a period of several weeks. Scale bars = 1 cm. Unpaired t tests were performed. **J** Representative images of three different gastric cancer organoids transfected with JMJD3 overexpression vectors or control vectors for 2 weeks. Scale bars = 200 µm. The data are the means ± S.D. of three independent experiments. *p < 0.05; **p < 0.01; ***p < 0.001; ****p < 0.0001.

Given the critical role of cancer stemness in the development of chemotherapy resistance [34], this study aimed to assess the impact of JMJD3 on the stemness characteristics of gastric cancer cells. Specifically, we evaluated the potential regulatory influence of JMJD3 on the expression of key stemness markers, including OCT4, LIN28, SOX2, CD133, and CD44, which are associated with gastric cancer. Downregulation of JMJD3 resulted in a significant decrease in the levels of CD44, SOX2, OCT4, and LIN28 in gastric cancer cells (Supplementary Fig. S3A, B). Conversely, overexpression of JMJD3 led to a marked increase in the expression of these stemness markers (Fig. 3A–C). Flow cytometry analysis further confirmed that JMJD3 overexpression increased the population of CD133^high^ gastric cancer cells (Fig. 3D, E), whereas JMJD3 knockdown reduced the proportion of CD133^high^ cells (Supplementary Fig. S3C, D). Additionally, tumor sphere formation assay revealed that JMJD3 overexpression promoted tumor sphere growth in SNU-1 and NCI-N87 cell lines, while JMJD3 knockdown inhibited sphere formation in AGS and HGC-27 cell lines (Fig. 3F, G; Supplementary Fig. S3E, F). To further investigate JMJD3’s role in regulating stemness, nude mice were subcutaneously injected with cells. As shown in Fig. 3H, I, SNU-1-JMJD3 and NCI-N87-JMJD3 mice developed significantly larger tumors compared to controls. Moreover, to further evaluate the tumor-promoting potential of JMJD3, patient-derived gastric cancer organoid (PDO) models were established using tumor tissues from three gastric cancer patients. Lentivirus-mediated JMJD3 overexpression significantly enhanced the growth and size of PDOs, indicating increased self-renewal and proliferative capacities of cancer stem cells (Fig. 3J). These observations further support the critical role of JMJD3 in enhancing gastric cancer malignancy and stemness.

### JMJD3 upregulates ALOX5 expression via reducing H3K27 trimethylation

**Fig. 4:**
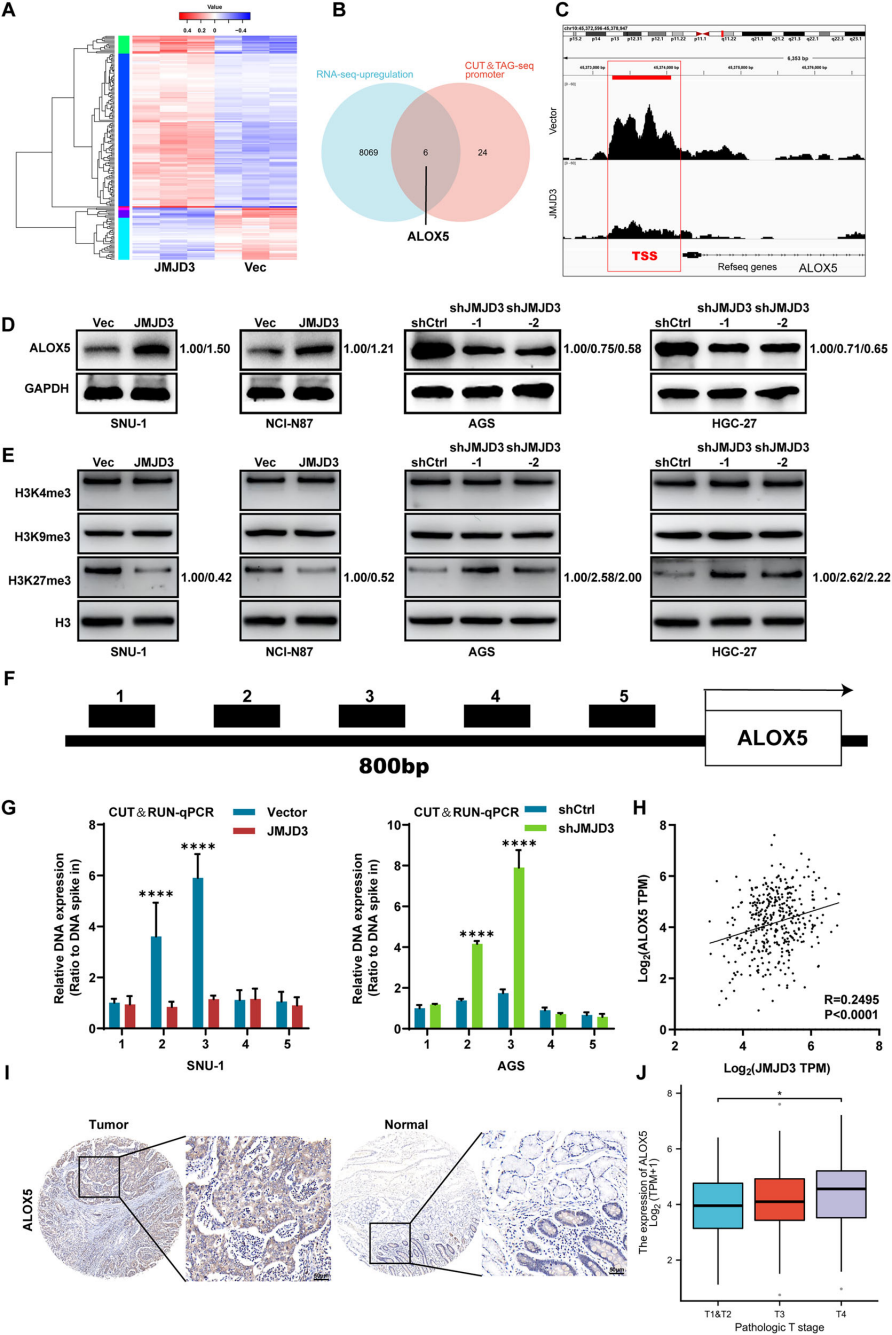
JMJD3 upregulates ALOX5 through reducing H3K27 trimethylation. **A** Supervised hierarchical clustering of the genes differentially expressed after JMJD3 overexpression in SNU-1 cells. **B** After overexpression of JMJD3, co-genes between up-regulated gene of Microarray analyses and the promoter region obtained in CUT&TAG-seq were obtained by Veen. **C** CUT&TAG-seq in SNU-1 cells visualized using IGV. TSS regions are shown in red graphs. **D** ALOX5 was measured in gastric cancer cells with JMJD3 overexpression or silencing, determined by western blot assay. **E** The abundance of H3 lysine methylation was assessed in gastric cancer cells with JMJD3 overexpression or silencing by western blot analysis using whole-cell lysate. **F** Schematic presentation of five regions relative to the ALOX5 transcriptional start site used as primers to test histone occupied abundance. **G** CUT& RUN-qPCR was performed to assess H3K27me3 occupancy in SNU-1-JMJD3, AGS-shJMJD3, or their control cells. **H** Correlation between JMJD3 and ALOX5 in TCGA database. **I** Expression of ALOX5 in gastric cancer detected by IHC. **J** Relationship between ALOX5 expression levels and patients’ T stage in the TCGA database. *p < 0.05; ****p < 0.0001 is based on the Student *t* test. The data are the means ± S.D. of three independent experiments.

To investigate how JMJD3 contributes to stemness and chemoresistance in gastric cancer, we performed gene expression profiling on SNU-1-JMJD3 and control cells. Microarray analyses identified several differentially expressed genes, including ALOX5, following JMJD3 overexpression (Fig. 4A). To further explore the genes regulated by JMJD3, we conducted CUT&TAG sequencing, which revealed several promoter regions that JMJD3 could bind to. Venn diagram analysis, combined with gene expression profiling, identified ALOX5 as a direct target of JMJD3 (Fig. 4B). Visualization of the CUT&TAG-seq results with the Integrative Genomics Viewer (IGV) showed that one region (marked red) of the ALOX5 promoter was demethylated by JMJD3 (Fig. 4C). Consistently, the protein levels of ALOX5 were significantly elevated in SNU-1-JMJD3 and NCI-N87-JMJD3 cells, while silencing JMJD3 in AGS and HGC-27 cells drastically reduced ALOX5 expression (Fig. 4D), suggesting that JMJD3 upregulates ALOX5 at transcription level.

To explore the link between JMJD3 activity and histone modifications in gastric cancer cells, we examined histone modifications following JMJD3 expression modulation. Analysis of histones H3K9me3, H3K4me3, and H3K27me3 revealed that only H3K27me3 was affected by JMJD3 expression. Ectopic JMJD3 expression reduced H3K27me3, while silencing JMJD3 enhanced this modification (Fig. 4E). Using CUT& RUN-qPCR assays, we further demonstrated that JMJD3 overexpression in SNU-1-JMJD3 cells led to decreased H3K27me3 levels at the ALOX5 promoter and whereas JMJD3 depletion resulted in increased H3K27me3 levels in AGS-shJMJD3 cells (Fig. 4F, G). This finding was consistent with data from the TCGA database, showing a correlation between JMJD3 and ALOX5 expression (Fig. 4H) while the expression of ALOX5 was also highly correlated with the pathological T stage in patients (Fig. 4J). IHC analysis also revealed elevated ALOX5 expression in gastric cancer tissues (Fig. 4I). These results suggest that JMJD3 promotes ALOX5 transcription in gastric cancer cells by reducing H3K27me3 at the alox5 promoter.

### JMJD3 maintains gastric cancer cell stemness by upregulating ALOX5

**Fig. 5:**
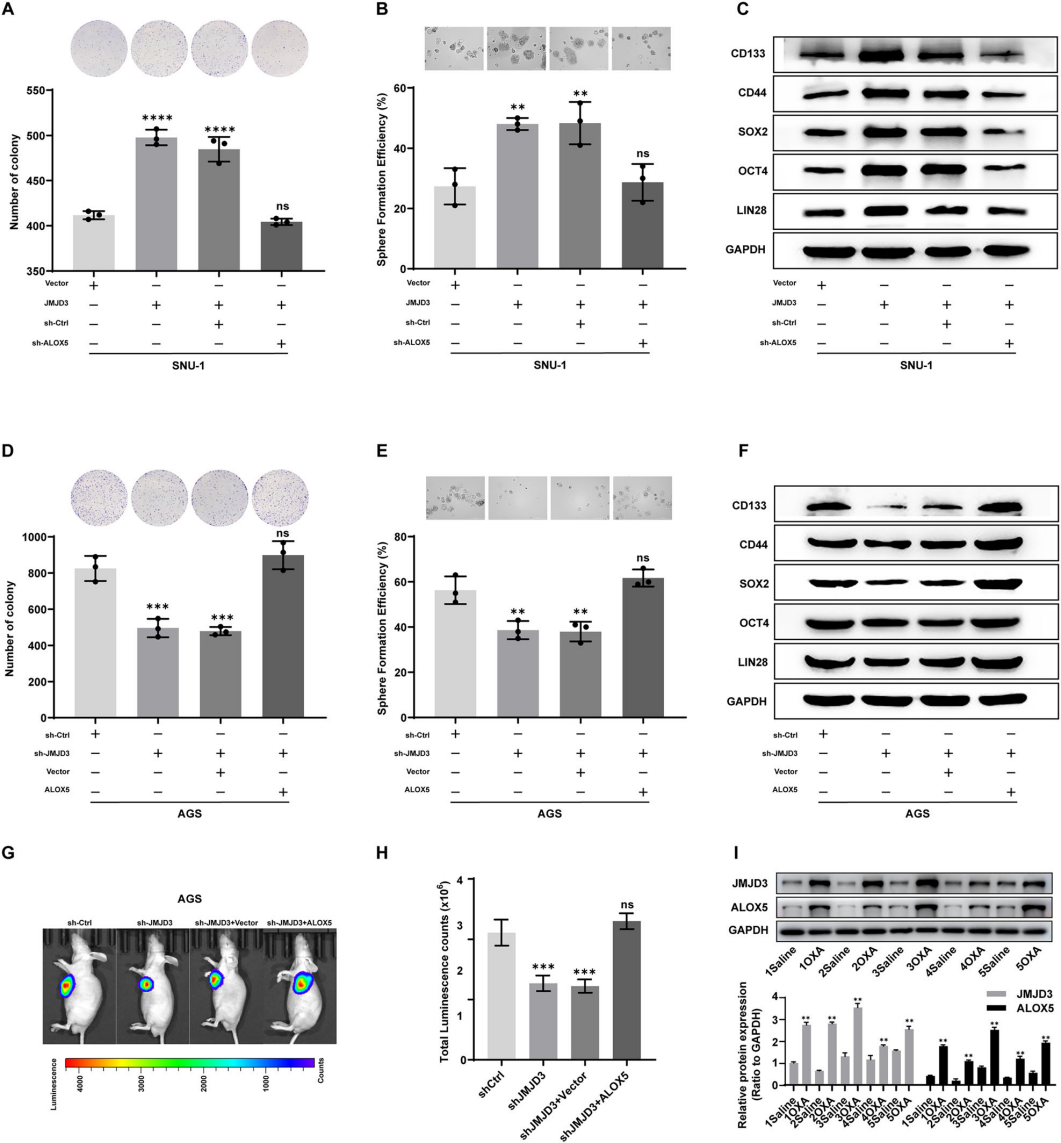
JMJD3 maintains gastric cancer cell stemness by upregulating ALOX5. **A** Colony formation. **B** Sphere formation. **C** Western blot of SNU-1 cells transfected with vector, JMJD3, sh-Ctrl, and/or sh-ALOX5. Expression of CD133, CD44, SOX2, OCT4 and LIN28 were measured. GAPDH was used as control. **D** Colony formation. **E** Sphere formation. **F** Western blot of AGS cells transfected with sh-Ctrl, sh-JMJD3, vector, and/or ALOX5. Expression of stemness markers were measured. **G** Representative ventral view images and its quantification (**H**) of bioluminescence. Scale bars = 200 µm (**B**, **E**). **I** Western blot analysis of JMJD3 and ALOX5 protein expression in tumors from the P3 generation of five patient-derived xenograft (PDX) models. Ordinary one-way ANOVA were performed. The data are shown as the means ± S.D. **p < 0.01; ns no significance.

To assess whether ALOX5 mediates the oncogenic role of JMJD3 in gastric cancer tumorigenesis, we first investigated if the oncogenic effect of JMJD3 overexpression could be attenuated through ALOX5 depletion. Stable SNU-1 cells expressing shRNAs targeting ALOX5 and JMJD3 expression vectors were generated. As anticipated, ALOX5 depletion effectively inhibited the colony forming capability enhanced by JMJD3 overexpression (Fig. 5A). Consistent results were obtained from sphere formation assay, where ALOX5 depletion reduced the sphere-forming capacity of SNU-1 cells overexpressing JMJD3 to base level (Fig. 5B). Additionally, as shown in Fig. 5C, the loss of ALOX5 reduced the expression of stemness markers. In vivo studies further revealed that ectopic ALOX5 expression in AGS cells reversed the tumor suppressive effect caused by JMJD3 deficiency (Fig. 5D–F). Bioluminescence imaging demonstrated that the reduction of tumor sizes and growth rate in JMJD3-deficient AGS xenografts was counteracted by ectopic ALOX5 expression (Fig. 5G, H). These results, consistent with in vitro findings, indicate that ALOX5 upregulation mediates the oncogenic function of JMJD3 in gastric cancer. To further support our conclusion, we analyzed protein expression in preserved tumor samples from the P3 generation of the OXA-resistant PDX models used in Fig. 1A. Western blot analysis revealed concurrent upregulation of JMJD3 and ALOX5 in OXA-treated tumors compared with their matched saline-treated controls (Fig. 5I). These findings not only reinforce the mechanistic axis identified in our cell line models but also highlight the clinical relevance of the JMJD3–ALOX5 pathway in chemoresistant gastric cancer.

### JMJD3 deletion in the mouse gastric epithelium inhibits tumor development

**Fig. 6:**
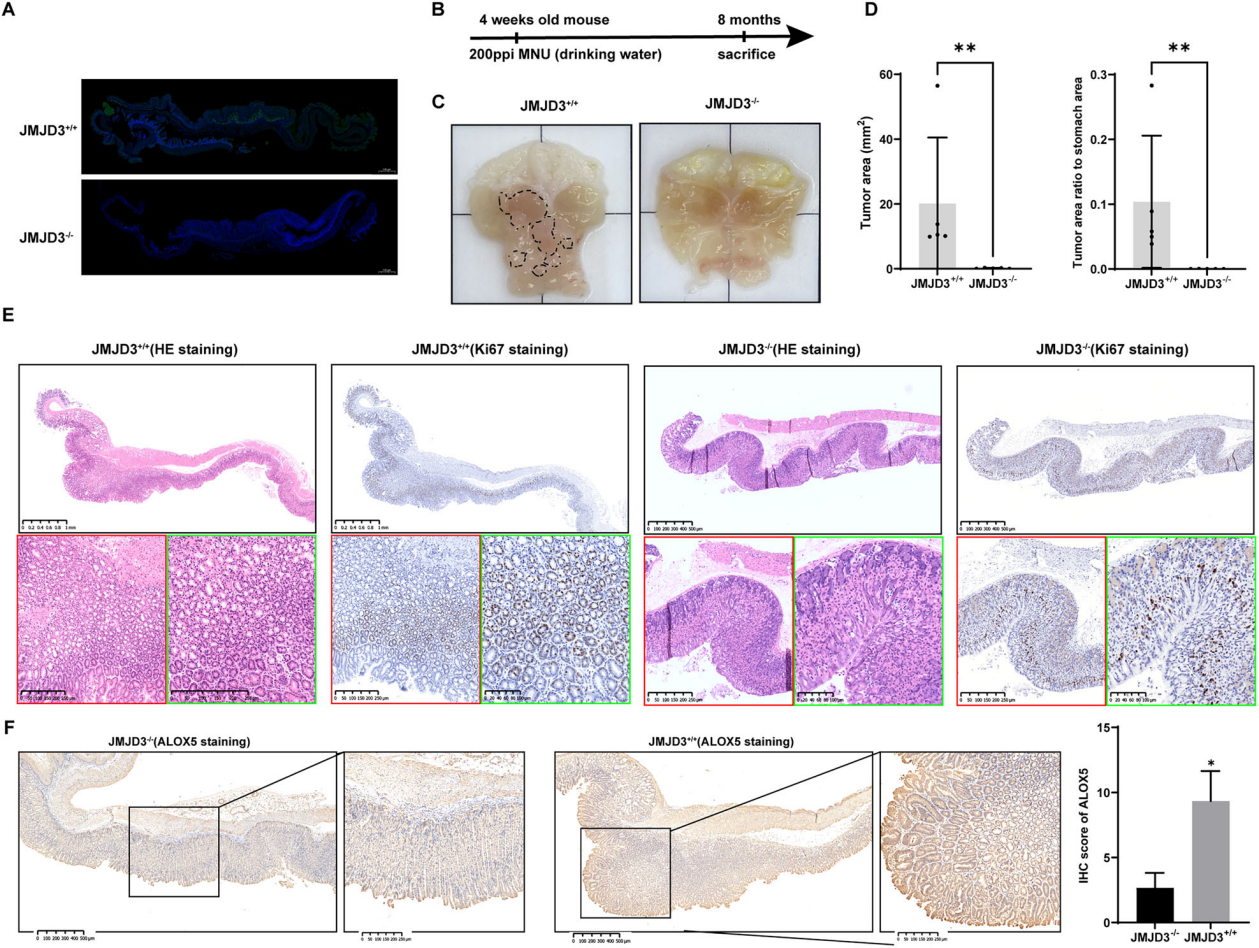
Knocking out JMJD3 in mouse gastric epithelium inhibits tumor development. **A** Immunofluorescence staining of JMJD3 in the gastric epithelium of JMJD3^f/f^; Tff1cre mice and control mice. **B** Flowchart of MNU-induced in situ gastric cancer in mice. **C** Tumor formation after MNU induction of in situ gastric cancer in JMJD3^+/+^ and JMJD3^−^^/−^ mice, with the circled areas indicating solid tumors protruding from the stomach. The border measures 2 cm. **D**, **E** HE and Ki67 staining results of gastric longitudinal sections from JMJD3^+/+^ and JMJD3^−^^/−^ mice, with JMJD3^+/+^ mice showing significant high-grade intraepithelial neoplasia. **F** AlOX5 staining results of gastric longitudinal sections from JMJD3^+/+^ and JMJD3^−^^/−^ mice, with JMJD3^+/+^ mice express more ALOX5. The data are shown as the means ± S.D. **p < 0.01.

To investigate the tumor-promoting role of JMJD3 in gastric cancer (GC), we utilized an MNU-induced GC model in mice with JMJD3 knocked out specifically in the gastric epithelium (Fig. 6A). After 8 months of MNU induction, the control group developed prominent gastric adenocarcinoma (Fig. 6B–E), while the JMJD3 knockout (KO) group either failed to develop visible tumors or only exhibited microscopically small tumors (< 1 mm²) (Fig. 6B, C). In histological analysis, the JMJD3-KO group displayed only epithelial thickening or intraepithelial neoplasia (Fig. 6E). Moreover, mice with intact JMJD3 in the gastric epithelium not only developed carcinoma in situ under MNU induction, but also showed significantly higher expression of ALOX5 in the gastric epithelium compared to the KO group (Fig. 6F). These findings indicate that JMJD3 promotes the development of gastric adenocarcinoma in vivo and further support JMJD3 as a potential therapeutic target in gastric cancer.

### JMJD3 confers ferroptosis sensitivity to gastric cancer cells

**Fig. 7:**
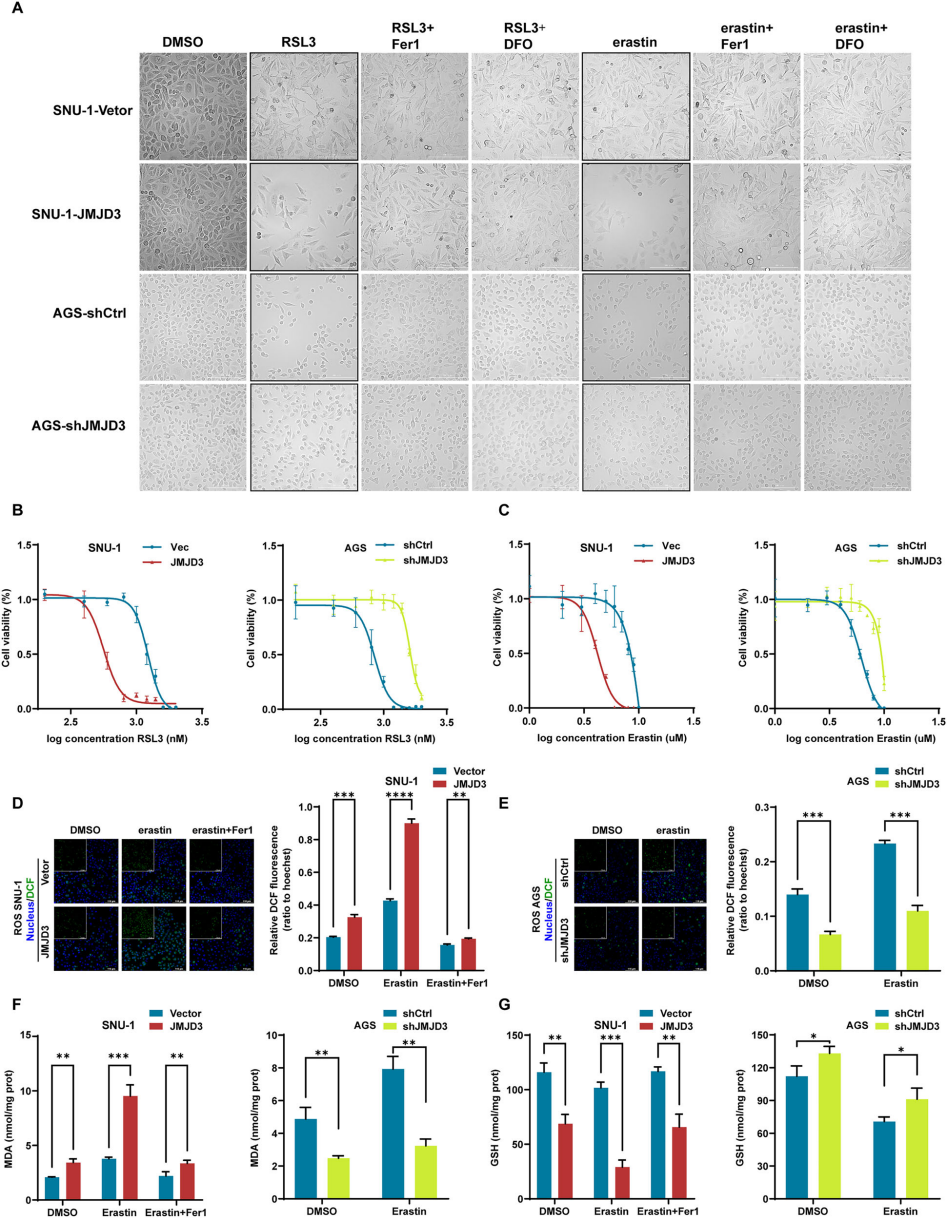
JMJD3 confers ALOX5-dependent ferroptosis sensitivity in gastric cancer cells. **A** Treatment of SNU-1-Vector/SNU-1-JMJD3/AGS-shCtrl/AGS-shJMJD3 with the ferroptosis inducer erastin and RSL3 inhibited the decrease of cells viability which can be rescued by ferroptosis inhibitors. **B**, **C** After treating SNU-1-Vector/SNU-1-JMJD3/AGS-shCtrl/AGS-shJMJD3 cells with RSL3 and erastin, we assessed cell viability to generate the drug IC50 curve, determining the half-maximal lethal concentration (LC50) of the ferroptosis inducer. **D**, **E** The level of ROS in SNU-1-Vector/SNU-1-JMJD3/AGS-shCtrl/AGS-shJMJD3 cells detected by confocal microscope. The results showed that in the erastin-treated group, JMJD3 rose the level of intracellular ROS, while ferroptosis inhibitor ferrostatin-1 reversed this process. **F** MDA content in SNU-1-JMJD3 cells significantly increased, and the difference was more significant after erastin treatment. After ferrostatin-1 treatment, the MDA content decreased again. **G** The GSH content in AGS-shJMJD3 cells increased, GSH content in AGS-shJMJD3 cells decreased, and the difference was more significant after erastin treatment and increased after ferrostatin-1 treatment. The data are shown as the means ± S.D. **p < 0.01; ***p < 0.001; ****p < 0.0001.

Our study reveals that JMJD3 regulates ALOX5, which has been shown to promote ferroptosis sensitivity in several studies [18, 19, 35]. We further analyzed the correlation between ALOX5 expression and ferroptosis-related genes in the TCGA database (Fig. S4A). Among the genes examined, ACSL4, NCOA4, and FTH1 showed significant correlation with ALOX5 (Fig. S4B–D). These findings prompted us to investigate whether JMJD3 influences ferroptosis sensitivity. We treated SNU-1 and AGS cells with ferroptosis inducers RSL3 and erastin, as well as inhibitors Ferrostatin-1 (Fer-1) and Deferoxamine (DFO) (Fig. 7A). Before imaging, non-adherent dead cells were gently washed away to better visualize the remaining adherent population. Under these conditions, SNU-1-JMJD3 cells exhibited significantly increased cell death compared to SNU-1-Vector cells, a phenotype that could be rescued by ferroptosis inhibitors. Similarly, in AGS-shCtrl and AGS-shJMJD3 cells, JMJD3 knockdown reduced sensitivity to ferroptosis inducers (Fig. 7B, C).

We further assessed reactive oxygen species (ROS) levels in gastric cancer cells using fluorescence microscopy. In the erastin-treated group, JMJD3 overexpression led to elevated cellular ROS levels, which were rescued by ferroptosis inhibitors (Fig. 7D, E). Additionally, we measured key ferroptosis markers, including glutathione (GSH) and the lipid peroxidation product malondialdehyde (MDA). The results revealed that MDA levels increased with higher JMJD3 expression, while GSH levels decreased, with these differences being more pronounced after erastin treatment (Fig. 7F, G). These findings suggest that in chemoresistant gastric cancer cells, JMJD3 not only enhances stemness but also sensitizes cells to ferroptosis, providing a potential therapeutic strategy to improve treatment efficacy.

## Discussion

OXA is a first-line chemotherapy drug for gastric cancer, and its mechanisms of resistance are multifaceted, including increased drug efflux, evasion of apoptosis, augmentation of autophagy, acquisition of stem cell-like nature, resistance conferred by tumor microenvironment, epigenetic changes, and redox imbalance. This study focuses on the development of cancer stem cell traits associated with resistance to OXA. Several molecular markers, such as CD133, CD44, OCT4, SOX2, and LIN28, have been used to define GCSCs. Like other stem cell types, GCSCs exhibit high expression of aldehyde dehydrogenase and multidrug resistance genes, with CD133 being the most prominent stemness marker [36, 37]. GCSCs typically acquire enhanced DNA repair capacity and are resistant to hypoxia and apoptosis, which allows them to better withstand platinum-based chemotherapy [38]. Our study reveals a critical role of JMJD3 in maintaining gastric cancer stem cell characteristics. Overexpression of JMJD3 led to upregulation of stemness markers, exemplified by CD133, and functional increase in stemness in gastric cancer cells. Furthermore, enhanced stemness in gastric cancer cells correlates with increased resistance to OXA, while silencing JMJD3 led to the opposite effect. In mice with gastric epithelial-specific JMJD3 knockout, the tumorigenic potential of MNU was markedly reduced. Additionally, we found that JMJD3 regulates ALOX5 overexpression in gastric cancer by directly binding to the alox5 promoter region and demethylating histones. Knock down of ALOX5 decreased the chemoresistance conferred by JMJD3. Thus, JMJD3 promotes stemness and chemotherapy resistance in gastric cancer cells by upregulating alox5 transcription. These findings suggest that elevated JMJD3 levels in gastric cancer patients may predict increased chemotherapy resistance and poor overall survival. The results shown here are consistent with the reports documenting the oncogenic role of JMJD3 in other types of malignancy [25, 39, 40].

Although ALOX5 is best known for its role in lipid peroxidation and ferroptosis regulation, evidence also suggests its involvement in cancer stemness. A previous study demonstrated that Alox5 is essential for the maintenance and leukemogenic potential of leukemia stem cells in chronic myeloid leukemia [41]. While similar mechanisms have not been fully established in solid tumors, our findings that ALOX5 upregulation enhances gastric cancer stemness markers and sphere formation suggest a comparable role in maintaining CSC-like phenotypes. It is plausible that ALOX5, through its lipid metabolites, may activate signaling pathways that support tumor self-renewal and therapy resistance.

Previous studies have shown that the high expression of ALOX5 is closely associated with ferroptosis sensitivity [42, 43], and ferroptosis is also closely linked to chemoresistance in cancer cells [14, 19, 44, 45], with chemoresistant cancer cells being more susceptible to GPX4 inhibition. In this study, we found that after developing resistance to oxalipatin, gastric cancer cells exhibit elevated levels of JMJD3, which mediates the upregulation of ALOX5 and allows the cells to survive and proliferate in the presence of OXA, accompanied by higher levels of reactive oxygen species (ROS). However, this JMJD3-mediated resistance to OXA also renders the cancer cells susceptible to ferroptosis inducers. The iron homeostasis system within these cells is more prone to collapse under external intervention, offering a novel therapeutic approach to overcoming chemoresistance in gastric cancer.

Several marketed drugs have been shown to induce ferroptosis in cancer cells. For example, sulfasalazine has demonstrated ferroptosis-inducing effects in multiple myeloma in a clinical trial (NCT04205357) [46]. The oral hypoglycemic agent has been shown to reduce the protein stability of SLC7A11, thereby increasing intracellular Fe²⁺ and lipid ROS levels, which induces ferroptosis [47]. Artesunate can induce ferroptosis in cancer cells through the activation of ferritinophagy [48, 49]. These drugs may potentially be used in combination with chemotherapeutic agents to enhance treatment efficacy, providing another promising therapeutic option for patients with chemoresistance gastric cancer characterized by elevated JMJD3 levels.

## Supplementary information

Supplementary Figures and Tables

Original Western Blots

Reproducibility checklist

## Data Availability

Most data generated or analyzed during this study are available within the article and its supplementary data files. Some of the data analyzed in this study were derived from the Gene Expression Omnibus (GEO) under accession numbers GSE113255 and GSE66229. In addition, the raw RNA sequencing and CUT&Tag sequencing data generated in this study have been deposited in the Science Data Bank (ScienceDB; https://www.scidb.cn/en) and are publicly accessible at the following links: RNA-seq: https://www.scidb.cn/s/73UrIj CUT&Tag-seq: https://www.scidb.cn/s/6RJnYb.
