# Supplementary material for: JMJD3 upregulates ALOX5 to drive malignancy and concomitant ferroptosis sensitivity in gastric cancer
Source: Cell Death Dis. 2025 Nov 3;16(1):782. doi: 10.1038/s41419-025-08020-1 (PMC12583487; doi:10.1038/s41419-025-08020-1)
Supplement: Supplementary file 1 — Supplementary Figures and Tables [file 41419_2025_8020_MOESM1_ESM.docx]

**Supplementary Materials for**

**JMJD3 Upregulates ALOX5 to Drive Malignancy and Concomitant Ferroptosis Sensitivity in Gastric Cancer**

**This PDF file includes:**

**Supplementary Figure 1 to 4**

**Supplementary Table 1 to 3**

**Supplementary Figure 1**

**
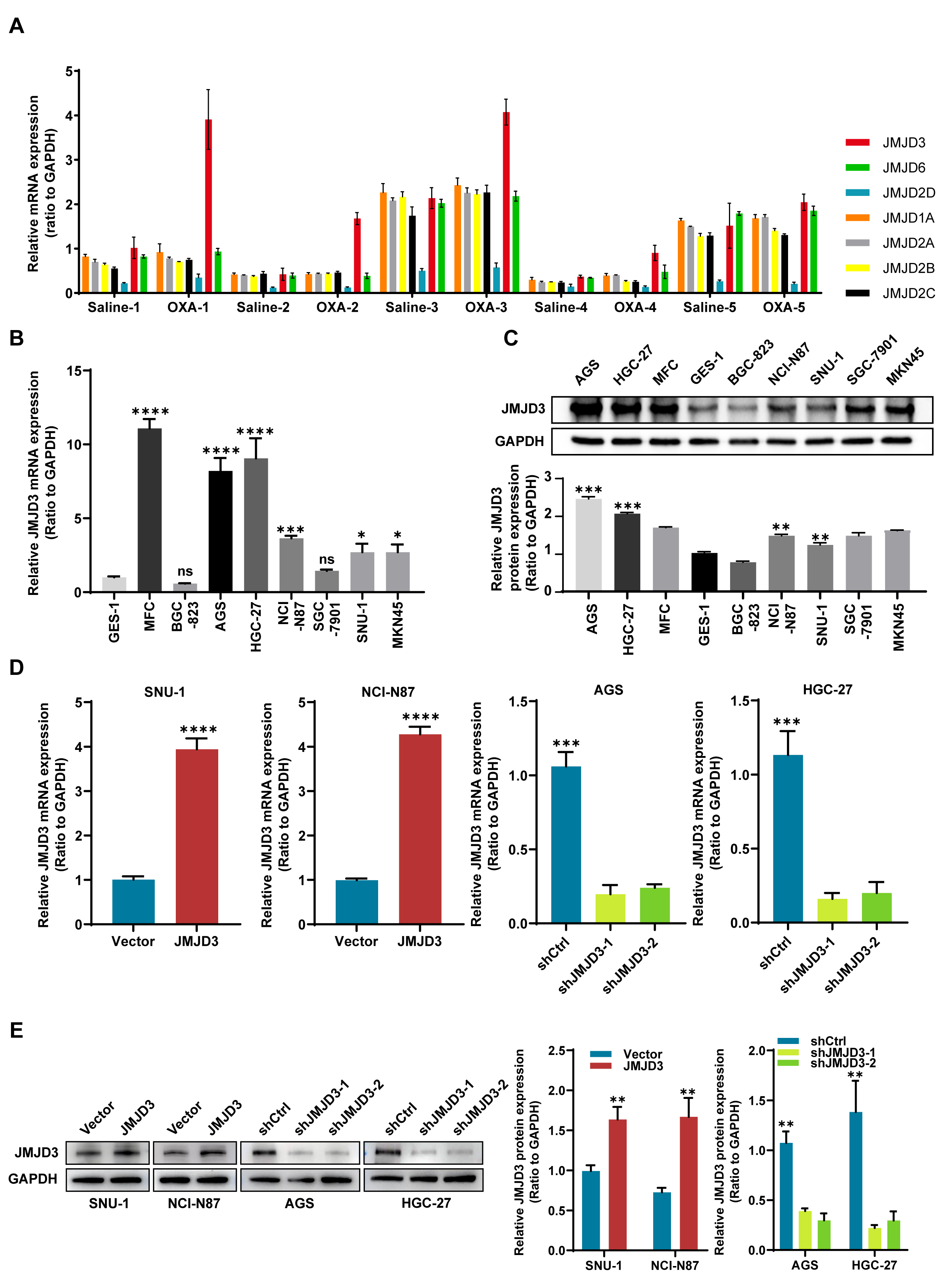
**

**Expression and manipulation of JMJD3 in gastric cancer.**

**A** mRNA expression levels of selected histone demethylases in PDX models. JMJD3 was significantly upregulated in oxaliplatin-resistant PDX tumors (P3 generation) compared to saline-treated controls. JMJD3 expression in gastric cancer cell lines compared to the normal human gastric epithelial cell line GES-1, assessed by qPCR (**B**) and Western blotting (**C**). JMJD3 was found to be highly expressed in AGS and HGC-27, but relatively low in SNU-1 and NCI-N87 cells. Validation of JMJD3 overexpression and knockdown efficiency in SNU-1, NCI-N87, AGS, and HGC-27 cell lines by qPCR (**D**) and Western blotting (**E**), confirming successful gene manipulation. Data are presented as means ± S.D. from three independent experiments. *p < 0.05; ***p < 0.001; ****p < 0.0001; ns, not significant.

**Supplementary Figure 2**

**
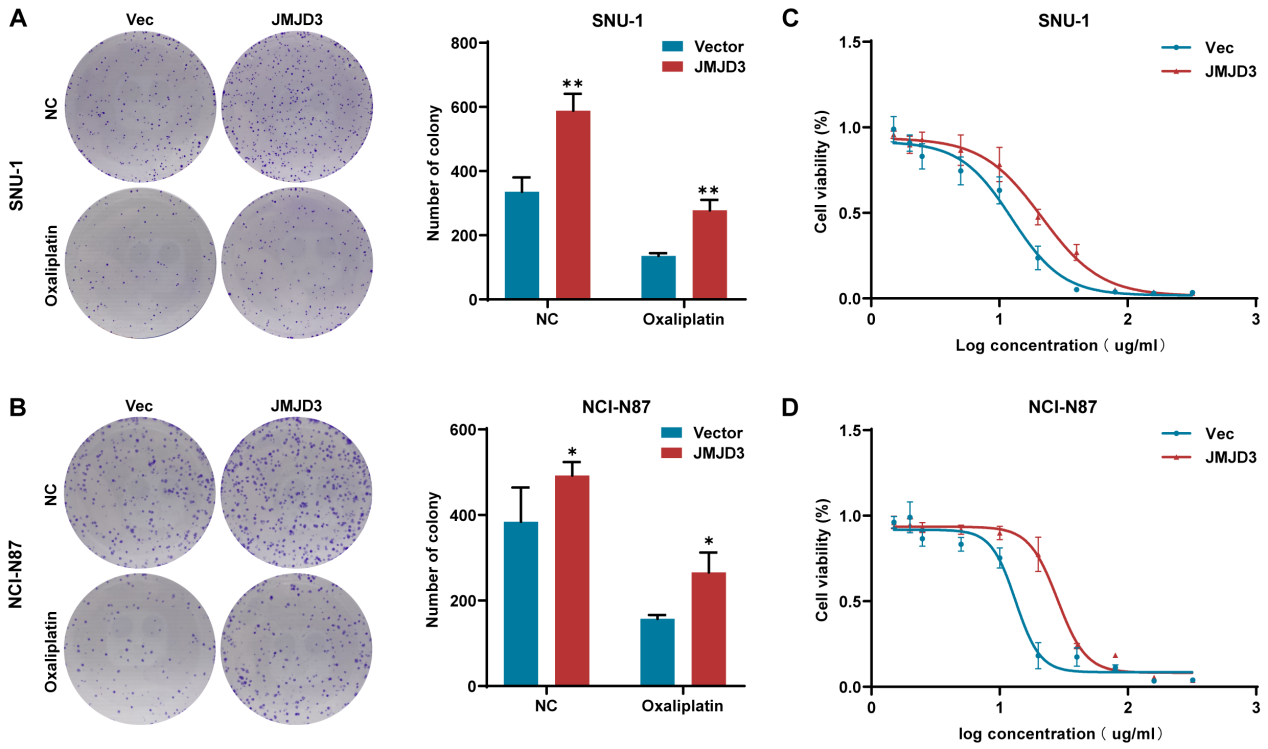
**

**JMJD3 suppresses the chemosensitivity of gastric cancer cells to oxaliplatin.**

Overexpression of JMJD3 enhanced the clonogenic ability (**A**, **B**) and tolerance of oxaliplatin (**C**, **D**) in gastric cancer cells. The data are the means ± S.D. of three independent experiments. * p<0.05; ** p<0.01;

**Supplementary Figure 3**

**
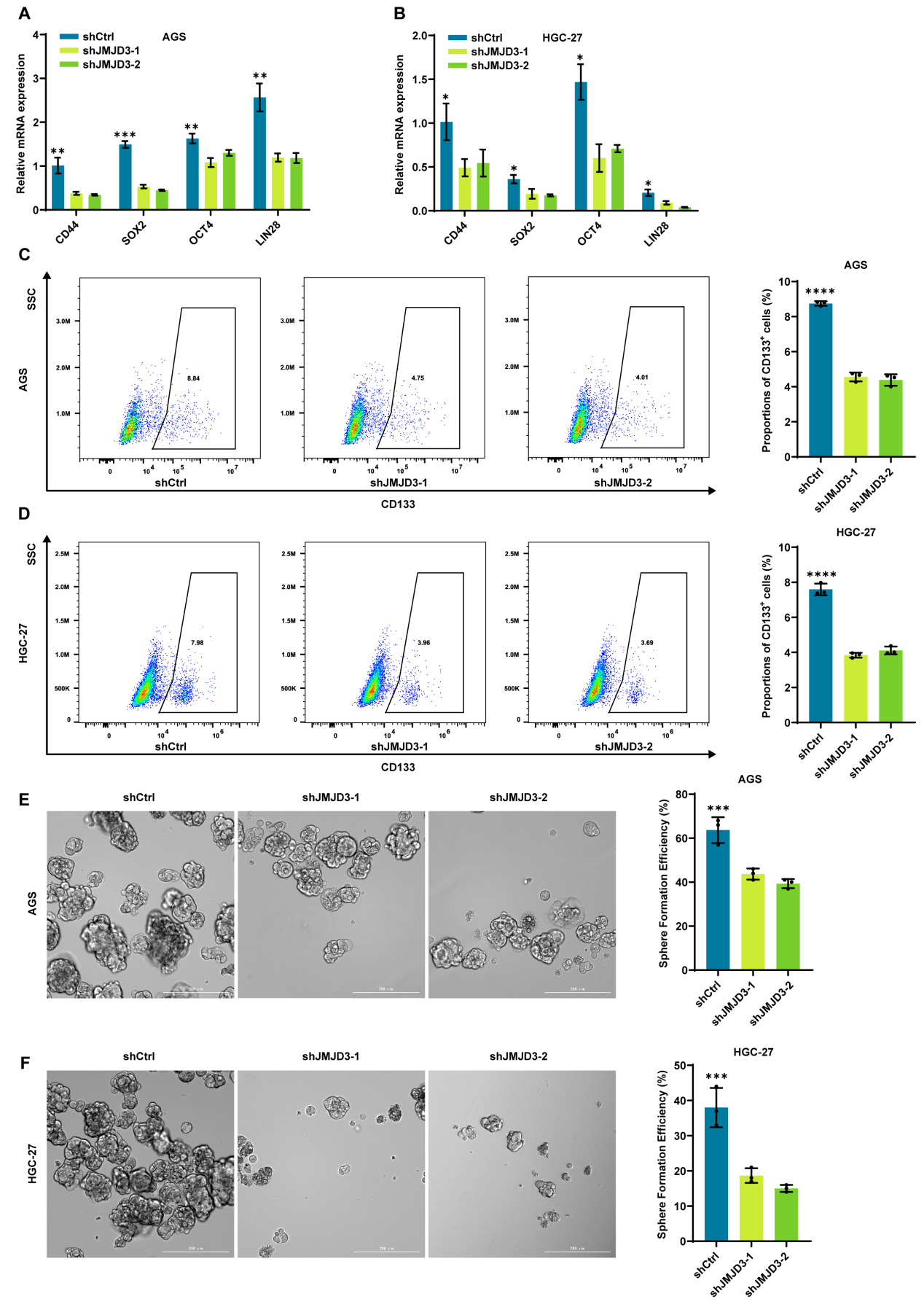
**

**Knockdown of JMJD3 reduces the stemness of gastric cancer cells.**

**A**, **B** The mRNA expression levels of CSC markers**. C**, **D** Flow cytometry showed that knockdown of JMJD3 can effectively reduce the number of CD133-positive cells in gastric cancer cells. **E**, **F** Knockdown of JMJD3 can limit the spheroidal ability of gastric cancer cells. Scale bars = 200 µm. The data are the means ± S.D. of three independent experiments. *** p<0.001; **** p<0.0001.

**Supplementary Figure 4**

**
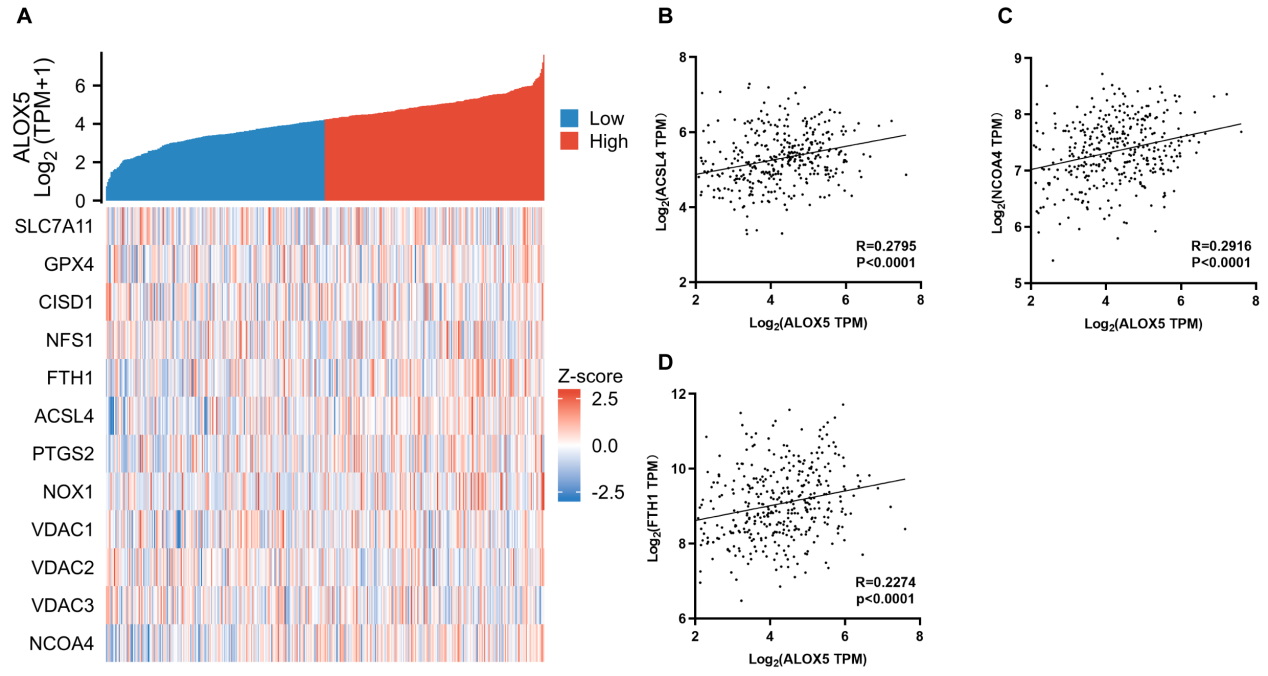
**

**ALOX5 is associated with ferroptosis.**

**A** Heatmap of the correlation between ALOX5 and various ferroptosis-related genes in the TCGA database. **B**, **C**, **D** Correlation of ACSL4, NCOA4, and FTH1 with ALOX5 in the TCGA database.

**Supplementary Table 1: primer sequences used in the study**

| JMJD3 | F: ATGCCAAGGTGAAAGGGAAGTTT  R: TCCCGTTTGCTCTCCAGATAGAT |
| --- | --- |
| ALOX5 | F: CGACTACATCTACCTCAGCCTCG  R: GTATGAATCCACCGCGCCAC |
| CD133 | F: TAGGACAGATTTGGATGG  R: TGATACCTGCTACGACAGT |
| CD44 | F: TTCAGGAGGTTACATCTTTT  R: TCTGTCTGTGCTGTCGGT |
| SOX2 | F: CCATGCACCGCTACGACG  R: TGGGAGGAAGAGGTAACCACAG |
| OCT4 | F: GGTATTCAGCCAAACGAC  R: CTTCCTCCACCCACTTCT |
| LIN28 | F: TCTGTAAGTGGTTCAACGTGCG  R: CATGTGCAGCTTACTCTGGTGC |
| CUT＆RUN-1 | F: TGAGGAGATGCTACAGGGACT  R: GCACACAGTCGCGTGTATTG |
| CUT＆RUN-2 | F: CAATACACGCGACTGTGTGC  R: CCTGGAAGGGTGTGGAGAAG |
| CUT＆RUN-3 | F: CCTTCTCCACACCCTTCCAG  R: TTCTTCACTGGCTGGACCTG |
| CUT＆RUN-4 | F: CAGGTCCAGCCAGTGAAGAA  R: CCTCTGCTCTCCCCAAGTTC |
| CUT＆RUN-5 | F: AGTGGCACTGAGAACTTGGG  R: CCACCCCTCATCCATTCGTT |
| DNA Spike in | F: GCCTTCTTCCCATTTCTGATCC  R: CACGAATCAGCGGTAAAGGT |
| GAPDH | F: CTCTGCTCCTCCTGTTCGAC  R: GCGCCCAATACGACCAAATC |

**Supplementary Table 2: Antibodies for western blot**

| Antibody name | Source | Item number |
| --- | --- | --- |
| JMJD3 | CST | 3457s |
| CD133 | Abcam | ab19898 |
| CD44 | Abcam | ab243894 |
| SOX2 | Abcam | ab137385 |
| OCT4 | Abcam | ab181557 |
| LIN28 | Abcam | ab279647 |
| Histone-H3 | Proteintech | 17168-1-AP |
| ALOX5 | Proteintech | 10021-1-Ig |
| H3K4me3 | CST | #9751 |
| H3K9me3 | CST | #13969 |
| H3K27me3 | CST | #9733 |
| GAPDH | Beyotime | AG109 |

**Supplementary Table 3：Summary of JMJD3 expression with clinicopathologic features of gastric cancer**

| **Variables** | **Cases**  **(n)** | **JMJD3 expression** | |  | **P Value** |
| --- | --- | --- | --- | --- | --- |
|  |  | **Low 45** | **High 45** |  |  |
| **Age** | | | | | |
| ＞60 | 46 | 20 | 26 |  | 0.206 |
| ≤60 | 44 | 25 | 19 |  |  |
| **Gender** | | | | | |
| Female | 41 | 17 | 24 |  | 0.138 |
| Male | 49 | 28 | 21 |  |  |
| **Tumor differentiation** | | | | | |
| Well/ Moderate | 32 | 14 | 18 |  | 0.378 |
| Poor | 58 | 31 | 27 |  |  |
| **Tumor size(cm)** | | | | | |
| ＜3.5 | 35 | 24 | 11 |  | 0.005* |
| ≥3.5 | 55 | 21 | 34 |  |  |
| **Lymph node invasion** | | | | | |
| Present | 49 | 22 | 27 |  | 0.290 |
| Absent | 41 | 23 | 18 |  |  |
| **TNM stage** | | | | | |
| I-II | 55 | 33 | 22 |  | 0.017* |
| III-IV | 35 | 12 | 23 |  |  |
| **Helicobacter pylori infection** | | | | | |
| YES | 48 | 29 | 19 |  | 0.035* |
| NO | 42 | 16 | 26 |  |  |

*<0.05
