# Supplementary figures and images for: JMJD3 upregulates ALOX5 to drive malignancy and concomitant ferroptosis sensitivity in gastric cancer
Source: Cell Death Dis. 2025 Nov 3;16(1):782. doi: 10.1038/s41419-025-08020-1 (PMC12583487; doi:10.1038/s41419-025-08020-1)

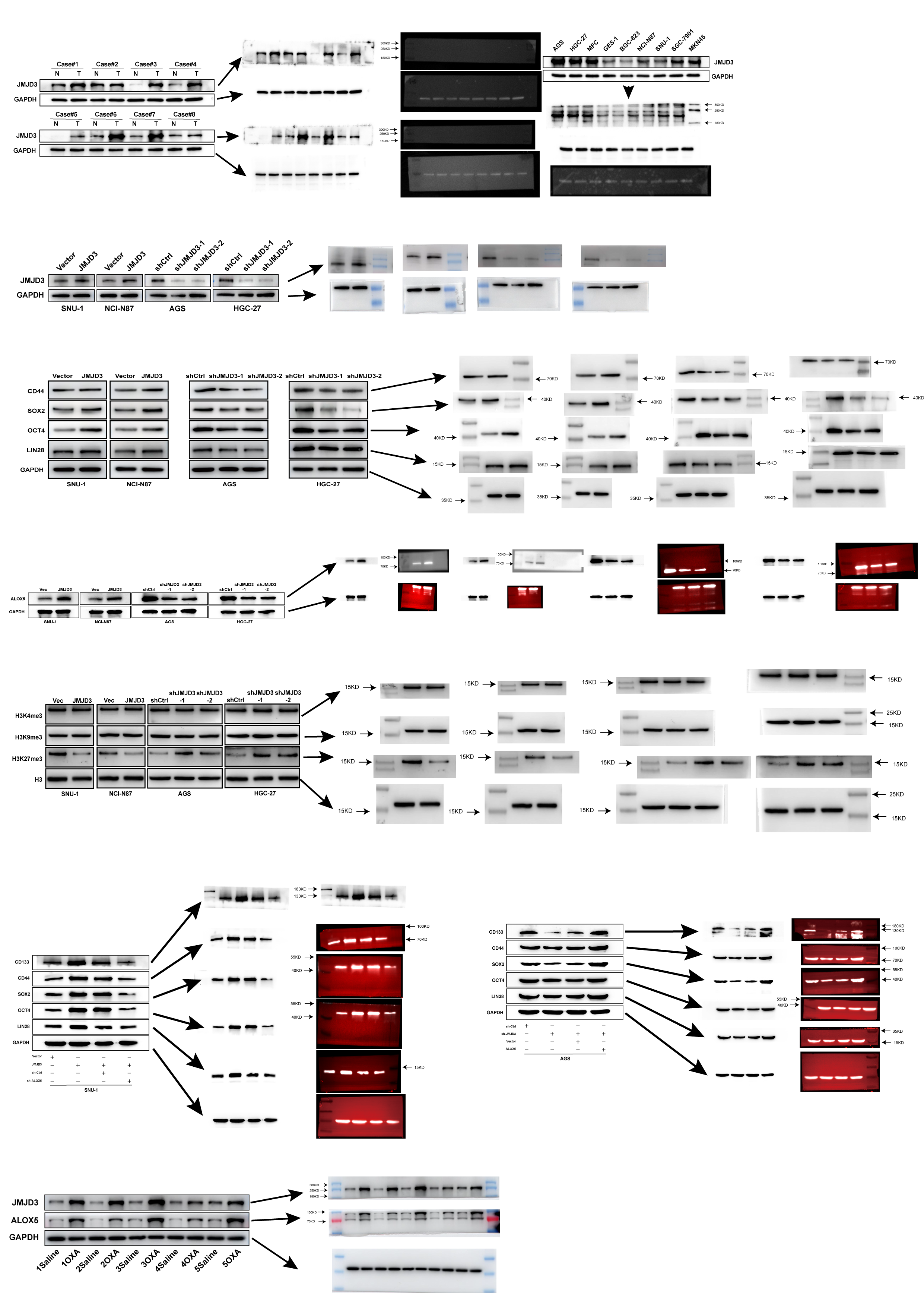

Supplement: Supplementary file 2 — Original Western Blots [file 41419_2025_8020_MOESM2_ESM.tif]
